# Acute effects of anodal transcranial direct current stimulation on maximal voluntary force, repeated maximal isometric contractions and corticospinal excitability in healthy young adults

**DOI:** 10.1007/s00421-026-06186-w

**Published:** 2026-03-02

**Authors:** David Colomer-Poveda, Irene Mera-González, Daniel Marcos-Frutos, Luna Botana-López, José Andrés Sánchez-Molina, Virginia López-Alonso, Gonzalo Márquez

**Affiliations:** 1 Sport Sciences Research Centre, Faculty of Education and Sport Sciences and Interdisciplinary Studies, Rey Juan Carlos University, Madrid, Spain; 2 Department of Physical Education and Sport, Faculty of Sports Sciences and Physical Education, University of A Coruna, A Coruña, Spain; 3 Department of Physical Education and Sport, Faculty of Sport Sciences, University of Granada, Granada, Spain; 4 MOBhE Group, Center of Higher Education Alberta Giménez (CESAG), Comillas Pontifical University, Palma, Spain

**Keywords:** TDCS, Non-invasive brain stimulation, Transcranial magnetic stimulation (TMS), Corticospinal excitability (CSE), Short-interval intracortical inhibition (SICI), Strength, Perceived effort.

## Abstract

**Purpose:**

The aim of this triple-blind, placebo-controlled study was to determine the effects of anodal transcranial direct current stimulation (a-tDCS) applied over the primary motor cortex (M1) and the dorsolateral prefrontal cortex (DLPFC) on maximal voluntary force, the ability to repeat maximal handgrip contractions, cortical and corticospinal excitability measured with transcranial magnetic stimulation (TMS), and rating of perceived effort.

**Methods:**

Forty-two healthy participants (19 women; 1 left-handed; 21.5 ± 1.4 years) completed three identical sessions differing only in the a-tDCS condition: 20 min of 2 mA a-tDCS over the M1, the DLPFC, and a sham session (30 s of real stimulation) in a randomized order. Maximal voluntary force, corticospinal excitability and intracortical inhibition and facilitation were assessed before (pre) and after (post) a-tDCS. Additionally, the number of maximal contractions performed until a 30% force loss in handgrip strength, and the overall perceived effort of the task were recorded following a-tDCS application.

**Results:**

The results showed no significant main effects or interactions in any behavioural, neurophysiological, or perceptual variables, beyond a small but consistent reduction from pre to post measurements in maximal voluntary force (-3.2%) across all sessions.

**Conclusions:**

These findings indicate that a-tDCS over M1 or DLPFC does not improve maximal voluntary force, endurance during repeated maximal contractions, perceived effort or associated cortical and corticospinal excitability in healthy young adults (all results *p* > 0.05). Accordingly, in healthy young adults, the present results do not support recommending a-tDCS as a performance-enhancing intervention for maximal or repeated maximal muscle efforts.

**Supplementary Information:**

The online version contains supplementary material available at 10.1007/s00421-026-06186-w.

## Introduction

Transcranial direct current stimulation (tDCS) is a portable and easily applicable device that generates a constant low intensity current (1–4 mA) through two or more electrodes, typically placed on the scalp. Due to its safety, non-invasive and painless nature (Stagg and Nitsche 2011), and its potential to modulate the properties of cortical neurons (Nitsche and Paulus 2000), the interest in the functional applicability of tDCS has increased during the last years. One of the fields that has accrued most of the research efforts is the study of the acute effects of a single session of tDCS applied before exercise on subsequent motor performance. Particularly, on muscle strength (Hendy and Kidgell 2014; Kan et al. 2013) and endurance capacity (Angius et al. 2016b; Lattari et al. 2018; Wang et al. 2022); two essential components of physical fitness.

Anodal-tDCS (a-tDCS) over the primary motor cortex (M1) has been used to increase maximal voluntary muscle force during single, unfatigued maximal voluntary dynamic or isometric contractions. This approach is typically motivated by evidence that M1 a-tDCS can increase the excitability of the cortical neurons (Nitsche and Paulus 2001), which has been hypothesised to improve the effectiveness of the motor command, leading to greater motor unit recruitment and force output. However, the effects of M1 a-tDCS on corticospinal excitability and, specially, their functional relevance for maximal voluntary force production are contradictory. While some studies found that a-tDCS may increase maximal voluntary force (Hazime et al. 2017; Hendy and Kidgell 2014; Hikosaka and Aramaki 2021), meta-analytical data shows no effect on performance during brief maximal isometric or dynamic muscle contractions (Alix-Fages et al. 2019). Furthermore, recent studies combining neurophysiological measurements with behavioural data to elucidate the mechanisms of action of a-tDCS report no effect on voluntary activation, corticospinal excitability (CSE) and maximal voluntary force (Giboin and Gruber 2018; Jonker et al. 2021; Kristiansen et al. 2022). Therefore, current evidence does not support using a-tDCS to enhance performance during single unfatigued maximal voluntary dynamic or isometric contractions (Kristiansen et al. 2022). However, the small sample size of the studies (*n* = 13–20), and the inherent variability in response to a-tDCS (López-Alonso et al. 2014) preclude firm conclusions.

Despite the apparent lack of effect on unfatigued maximal force production, an increase in CSE after M1 a-tDCS can influence performance during longer efforts, such as sustained or repeated maximal or submaximal muscle contractions until failure or a predetermined fatigue threshold (Alix-Fages et al. 2019; Flor et al. 2025). Several studies have reported an increase in the total number of repetitions performed during a moderate intensity resistance training session in back squat (Fortes et al. 2021), the number of burpees performed until exhaustion (Chen et al. 2024) or the time to task failure during a sustained knee extension contraction at 30% of the maximal voluntary contraction (MVC) force (Angius et al. 2016a). However, other studies found no effect of M1 stimulation on the ability to repeat high-intensity dynamic (Workman et al. 2020) or isometric (Giboin and Gruber 2018) muscle contractions. Notwithstanding, the effect of a-tDCS has been tested in other cortical areas like the dorsolateral prefrontal cortex (DLPFC), which merges cognitive and sensory signals during exercise, playing a crucial role in suppressing subjective fatigue (Grosprêtre et al. 2021; Robertson and Marino 2016). Although DLPFC stimulation has no effect on force production during brief efforts like jumping (Romero-Arenas et al. 2021) or sprinting (Alix-Fages et al. 2022), several studies have found increases in the number of repeated dynamic and isometric high-intensity muscle contractions performed and reduced ratings of perceived exertion (RPE) after DLPFC stimulation (Alix-Fages et al. 2020; Lattari et al. 2020; Rodrigues et al. 2022). Therefore, the interaction between the simulated cortical area and the type of task may determine the presence or absence of a-tDCS effects on performance. However, one remarkable question is that only few studies with low-sample sizes have directly compared the effects of a-tDCS over M1 or DLPFC on tasks of different nature (i.e. brief maximal efforts vs. repeated maximal contractions) while also including neurophysiological measurements to determine relationships between cortical modulation and performance outcomes (Cogiamanian et al. 2007; Kristiansen et al. 2022; Lampropoulou and Nowicky 2013). Furthermore, recent evidence suggests that tDCS has minor or no effects on CSE (Jonker et al. 2021; Van der Cruijsen et al. 2022), casting doubts on their relevance regarding its abovementioned functional and performance effects.

Therefore, the aim of the present study was to determine, in a handgrip model, the specificity of a-tDCS effects depending on the stimulation area (M1 or DLPFC) on two performance domains: (a) maximal force production during single unfatigued maximal isometric contractions and (b) the ability to repeat maximal isometric contractions until a predetermined force loss threshold (−30%) in a large sample size (*n* = 42). Additionally, we measured CSE and intracortical inhibition and facilitation with single- and paired-pulse transcranial magnetic stimulation (TMS). Although there is currently little evidence suggesting a direct relationship between TMS-derived measures and variations in the efficacy or efficiency of the central motor command, including those measures could help to better understand their role as potential mechanisms leading to behavioural changes in maximal brief or repeated force production after a-tDCS. We hypothesize that only M1 stimulation will increase maximal force production, accompanied by changes in corticospinal and intracortical excitability, while both M1 and DLPFC stimulation, will influence the ability to repeat maximal isometric contractions and reduce the rate of perceived exertion, resulting in a greater number of repetitions compared to a placebo (Sham) stimulation condition.

## Methods

### Participants

Healthy recreationally active (2–3 h/week of sports or aerobic training) university students (*n* = 42, 19 women; 1 left-handed; 21.52 ± 1.40 years; height: 168.40 ± 26.44 cm; weight: 68.31 ± 11.86 kg), with no reported contraindications to TMS and not currently taking any medications volunteered to participate in the study. None of the participants had previously undergone tDCS. Sample size was estimated a priori using G*Power 3.1.9.7 (F tests, repeated measures ANOVA, within factors) with a medium effect size (f = 0.25), an alpha level of 0.05, a desired power of 0.80, one group, and three measurements, which indicates a minimum sample size of 36 participants.

All the participants were informed about the experimental protocol and signed an informed consent form approved by the Research Ethics Committee of A Coruña-Ferrol (2022/309). Participants were instructed to refrain from strenuous physical activity, drinking alcoholic or caffeinated beverages, and maintain their daily sleep and diet habits for 48 h prior to any evaluation. The investigation was conducted in accordance with the latest version of the Declaration of Helsinki.

### Study design

**Fig. 1:**
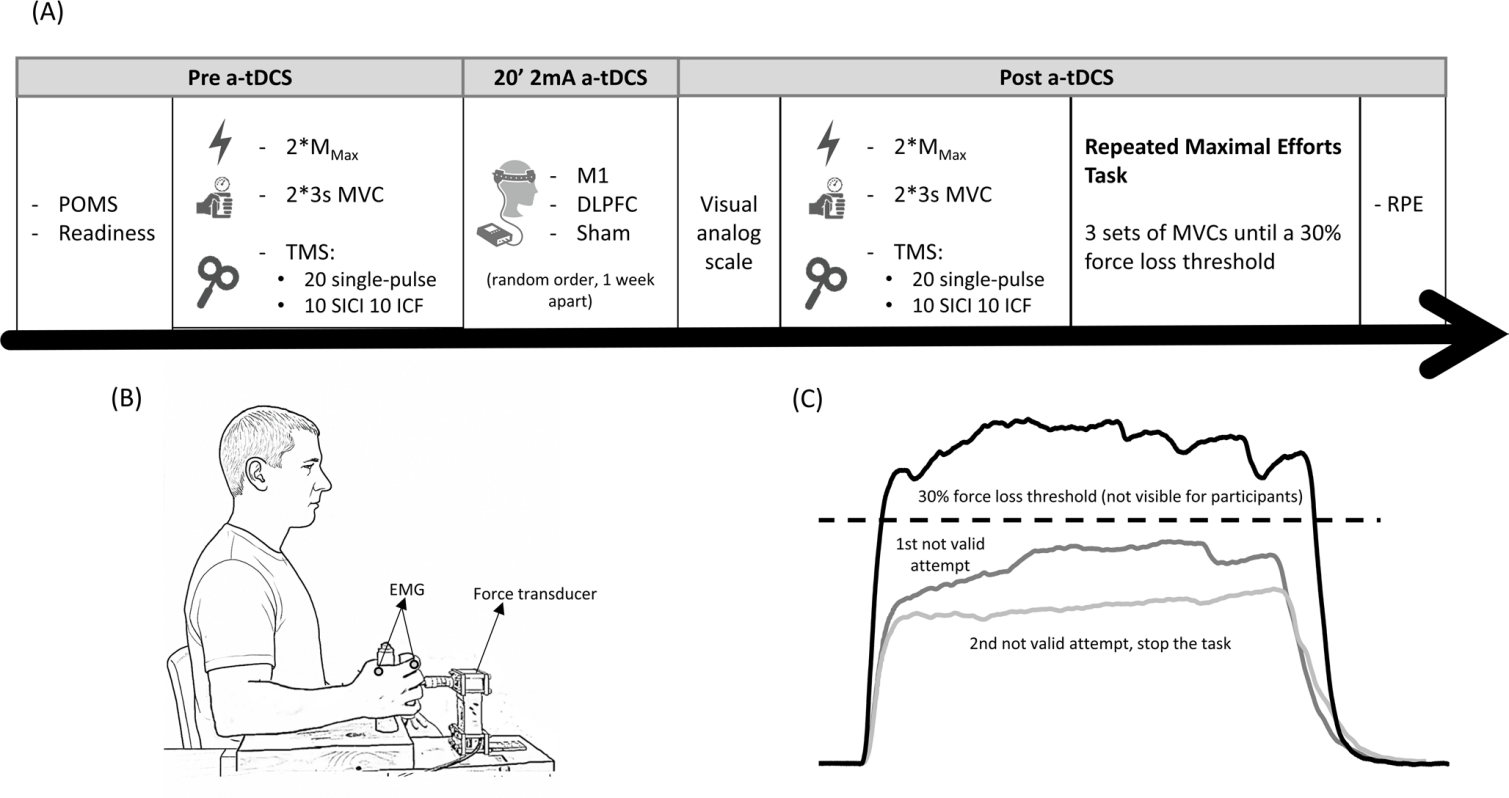
Schematic view of the experimental protocol (**A**). Experimental set-up (**B**). (C) Representation of the repeated maximal efforts task: participants performed sets of MVCs until a 30% force loss threshold was reached (dashed line, threshold not displayed to participants)

Each participant completed one familiarization session and three identical experimental sessions differing only in the a-tDCS condition. All the experimental sessions were separated by 5–7 days to allow for the washout of effects and were performed in a randomized crossover design. During the familiarization session participants tested all the procedures used during the experimental session including peripheral nerve stimulation, TMS, MVCs and the specific repetitions to task failure protocol to reduce any learning effect. At the beginning of the experimental sessions, the participants completed a Profile of Mood State questionnaire (POMS) and some questions about their general physical state (wellbeing questionnaire). Baseline measurements included peripheral nerve stimulation to obtain the maximum compound muscle action potential (M_Max_), single- and paired-pulse TMS at the first dorsal interosseous (FDI) hot-spot, and handgrip MVCs. These measurements were followed by 20 min of a-tDCS stimulation over M1, DLPFC or a placebo condition (Sham). After the a-tDCS stimulation period, baseline measurements were repeated, and a final fatigue task was performed to determine the effects of a-tDCS on the ability to perform repeated handgrip MVCs until task failure (i.e. inability to achieve 70% of the participant’s MVC in two attempts). Figure 1A illustrates the study design. The study had a triple-blind design where neither the participant, the evaluator nor the investigator performing the off-line data analysis knew the a-tDCS condition associated with each session.

### Set-up

Participants sat in a chair with the right forearm on an armrest, shoulders in adduction, elbow bent at 90º and the forearm in a neutral position (see Fig. 1B). In this position the right handgrip force was measured with a custom-made hand dynamometer using a commercial strain gauge (NL63, 200 kg; Digitimer, Welwyn Garden City, UK) connected to a Neurolog (Digitimer, Welwyn Garden City, UK) amplifier (Colomer-Poveda et al. 2023). Body placement and hand positions were standardized throughout the experiment. The fingers were fixed with tape to the dynamometer handles to keep a constant position of the hand. The force signal was amplified (x2500) and sampled (2 kHz) for offline analysis (Power 1401, Cambridge Electronic Design United Kingdom). After abrading and cleaning the skin with alcohol, superficial electromyography (EMG) was recorded from the right FDI with two pregelled Ag-AgCl surface electrodes, one positioned at the muscle belly of the FDI and the other at the proximal phalanx of the index finger. The EMG signal was amplified (x200), band-pass-filtered (10–500 Hz) and sampled (2 kHz) with a commercial wireless EMG (Noraxon Ultium; Noraxon LTD; USA) connected to a data acquisition interface and software for their storage and offline processing and analysis (Power 1401, Signal 6.06, Cambridge Electronic Design Ltd., Cambridge, UK).

## Experimental procedures

### Baseline assessment of wellbeing and mood

To assess the participants’ baseline state prior to the measurements, we administered the POMS short form (Spielberger 1972) and a wellbeing questionnaire (Hooper et al. 1995). This form is an abbreviated version of the POMS, consisting of 37 items that evaluate six mood dimensions: Tension-Anxiety, Depression-Dejection, Anger-Hostility, Vigour-Activity, Fatigue-Inertia, and Confusion-Bewilderment. Participants rate each item on a 5-point Likert scale ranging from 0 (“Not at all”) to 4 (“Extremely”). The scores for each dimension are summed, with higher scores indicating greater intensity of the corresponding mood dimension. The wellbeing questionnaire is used to determinate an individual’s mental state and preparedness for an upcoming task or intervention. Participants self-reported their sleep quality (1 = Insomnia, 5 = Acceptable, 9 = Very restorative), fatigue (1 = Very rested, 5 = Acceptable, 9 = Very fatigued), muscle soreness (1 = I feel great, 5 = Acceptable, 9 = Intense pain), and stress (1 = Very relaxed, 5 = Acceptable, 9 = Very stressed) using a 9-point scale (Hooper et al. 1995).

### Peripheral nerve stimulation

M_Max_ of the right FDI was obtained via single-pulse electrical stimulation (200 µs duration, DS7AH constant current stimulator, Digitimer, Welwyn Garden City, UK) through two adhesive pregelled Ag-AgCl surface electrodes positioned above the ulnar nerve just proximal to the wrist. The stimulation intensity was set at a 120% of the intensity needed to obtain a maximum M wave in the target muscle (range 21–160 mA).

### Voluntary maximum isometric contraction

After completing a standardized warm-up, all participants performed two 3-seconds MVCs with their right hand with 2 min of rest between attempts. During the MVCs, the researchers encouraged the participants, who also checked for variations in posture or simultaneous contractions of uninvolved muscles. Additionally, participants received visual feedback of their strength on a monitor in front of them during their run (Fig. 1B). This procedure, including the warm-up, was repeated before (pre) and after (post) 20 min of a-tDCS.

### Transcranial magnetic stimulation

Single- and paired-pulse TMS was delivered to the left M1 with a figure of eight-coil (70 mm diameter; stimulator: DuoMAG, Rogue Resolutions, Cardiff, UK) oriented with the handle pointing backward and at an angle of 45 degrees with respect to the midline. The coil was moved through the scalp to localize the FDI cortical representation (i.e., hot-spot), determined as the point where a known suprathreshold stimulation intensity produced the largest response, which was marked with a permanent marker. The participant’s resting motor threshold (rMT) was determined as the minimum intensity of stimulation that produced three out of five motor evoked potentials (MEPs) of a peak-to-peak amplitude of at least 50 µV. Once the hot-spot and the rMT were determined, a single block consisting of 20 single-pulses (120% rMT) and 20 paired pulses was obtained. Ten of the 20 paired-pulses were used to determine short intracortical inhibition (SICI) whereas the other ten were used to measure intracortical facilitation (ICF). Paired-pulses consisted in a conditioning stimulus and a test stimulus at an intensity of 80 and 120% of the rMT, respectively. The interstimulus interval was set to 3ms for SICI and 10ms for ICF. Pulses were separated by five seconds, and the order was randomized following previous studies (Colomer-Poveda et al. 2020).

### Transcranial direct current stimulation

Participants remained seated during the 20 min of a-tDCS. Stimulation intensity was set at 2 mA with a 30 s ramp-up and ramp-down. The stimulation was administered through a battery-powered continuous current stimulator device (TCT-Research neurostimulator, TransCranial Technologies, Hong Kong, China). A pair of surface electrodes (35 cm²) were placed within saline-soaked sponge pads to ensure conductivity. For the M1 condition, the anode was placed over the motor hot-spot determined by TMS. For DLPFC, the anode was placed 5 centimetres anterior to the hot-spot (Pascual-Leone et al. 1996). In all conditions, the reference electrode was positioned in the contralateral supraorbital fossa. For the Sham condition, half of the participants received the M1 configuration, while the other half received the DLPFC configuration. During the Sham condition, current was applied only for the first 30 s before being discontinued to mimic the initial sensations of active stimulation. Electrodes were secured using an adjustable strap to ensure stability. At the end of the 20–minute stimulation, participants rated their perception of itching, discomfort and burning sensations using a Visual Analog Scale (Russo et al. 2013) (only 36 out of the 42 rated their perceptions during all the experimental sessions).

### Repeated maximal isometric contractions task

After the administration of a-tDCS and the repetition of the measurements performed before the stimulation block (i.e. M_max_, MVC and TMS measurements), participants performed the repeated maximal isometric contractions task. This task was performed only once per session, at the end of the experimental protocol (Fig. 1). It involved performing 2-second maximal handgrip isometric contractions, interspersed with 2-second rest interval, repeatedly until task failure. Visual marks on the computer screen were used to pace the contraction and rest periods (2 s contraction – 2 s rest). Task failure was defined as the inability to achieve 70% of the participant’s MVC in two attempts, whether consecutive or not. The 30% force-loss threshold was chosen based on pilot testing, in which it provided enough repetitions per set while inducing substantial fatigue without excessive discomfort. Participants completed three sets of this task, with each set separated by a one-minute rest period. Participants received verbal encouragement throughout the task, and they were kept blinded to the force-loss threshold that determined task failure (30%). To maximize motivation and ensure consistent effort, a financial reward was offered to the three participants achieving the highest total number of repetitions across the three sets. After completing all three sets, participants reported their RPE using the Borg scale (6–20) (Borg 1982).

### Data analysis

The peak-to-peak amplitude values of M_Max_ and TMS single-pulses (MEPs) were measured for each participant. For the M_Max_, the average of the two recorded values was calculated. In the case of MEPs, any pulses falling outside ± 2 standard deviations (SDs) from the mean within each measurement block (pre or post) were excluded from the analysis. To account for peripheral-level variability and isolate CSE changes, pre MEP amplitudes were normalized to pre M_Max_, while post MEP amplitudes were normalized to post M_Max_ and expressed as MEP/M_Max_. Paired pulse measurements (i.e. SICIs and ICFs) were expressed as a percentage of the average MEP and subsequently averaged for further analysis. Pre-stimulus electromyographic (EMG) activity was analysed by calculating the root mean square (RMS) of the signal over a 200 ms interval preceding each electrical or magnetic stimulus to ensure all pulses were given at rest. Trials in which the RMS values fell outside ± 2 SDs of the mean for each measurement block were excluded from analysis to ensure data consistency. For TMS variables, only data from 41 participants was analysed, as TMS measurements were not successfully obtained in one participant. MVC was determined as the average peak force of two maximal attempts. For the repeated maximal isometric contractions task the number of successful repetitions performed in each set (Reps-Set1, Reps-Set2, and Reps-Set3) was recorded, along with the total number of repetitions completed across all three sets (Total-Reps) for each experimental session.

### Statistical analysis

Prior to conducting the statistical analyses, normality was assessed for all variables using the Shapiro-Wilk test to ensure the appropriate selection of parametric or non-parametric tests.

We determined baseline differences in mood and wellbeing with a one-way repeated measures analysis of variance (RM-ANOVA) or a Friedman test depending on data distribution. When significant differences were found, post-hoc comparisons with Bonferroni correction or Wilcoxon signed-rank tests with Bonferroni-adjusted p-values were performed to analyse pairwise differences between conditions. To determine baseline differences and the stimulation effect on repeated measures dependent variables (M_Max_, MEP/M_Max_, SICI, ICF, MVC), a two-way RM-ANOVA was conducted with normally distributed data, with condition (Sham, M1, DLPFC) and time (pre, post) as factors. If sphericity was violated (Mauchly’s test), degrees of freedom were corrected by Greenhouse-Geisser estimates of sphericity. When significant interactions or main effects were found, Bonferroni correction was applied to account for multiple comparisons in the post-hoc analyses. When data was not normally distributed, we used a non-parametric ANOVA-type test using the “nparLD” package in R (Noguchi et al. 2012).For variables measured only once per session after a-tDCS stimulation (i.e., Total-Reps, Reps-Set1, Reps-Set 2, Reps-Set 3 and RPE), a one-way RM-ANOVA or a Friedman test (depending on data distribution) was used to compare the three conditions. For parametric RM-ANOVAs, effect sizes were expressed as partial eta squared (ηp²), and approximate 95% confidence intervals were derived from the corresponding F statistics and degrees of freedom. For variables analysed with Friedman tests, effect sizes were expressed as Kendall’s W, with the 95% confidence intervals derived from the corresponding χ² statistics and sample size. For non-parametric ANOVA-type models fitted with the nparLD package, we report the relative treatment effects (RTE) and their approximate 95% confidence intervals for each level of the main factors (Time and Condition) in a table of the resources. Statistical significance was set at *p* < 0.05.

In addition to the main analyses described above, we performed a complementary cluster analysis. Due to the well-documented high inter-individual variability in the response to a-tDCS (López-Alonso et al. 2014), we classified participants as responders (R) or non-responders (NR) to a-tDCS based on changes in CSE. We considered as responders those participants who showed a positive percentage change between pre-MEP and post-MEP for each real stimulation condition (i.e., we obtained two separate clusters (R and NR) for the M1 and DLPFC conditions). After classifying participants as R and NR for each condition, we conducted a non-parametric ANOVA-type analysis with time (pre, post) and Group (R and NR) as factors for MVC, and with time (Set 1, Set 2, Set 3) and Group (R and NR) as factors for the number of repetitions performed during the task. These analyses were conducted separately for the M1 and DLPFC session data in order to determine whether participants whose CSE responded positively to a-tDCS also exhibited a positive influence of stimulation on behavioural outcomes. Additionally, due to the balanced sex distribution in the sample, we performed an exploratory analysis to investigate potential sex effects. For this purpose we added sex as a between-subject factor in the RM-ANOVA for normally distributed repeated-measures variables (M_Max_), and in the ANOVA-type models (nparLD) for non-normally distributed repeated measures (MEP/M_Max_, SICI, ICF, MVC) and single post-stimulation outcomes (Total-Reps, Reps-Set1, Reps-Set 2, Reps-Set 3 and RPE). When significant main effects or interactions were found with the non-parametric ANOVA-type test, a Wilcoxon signed rank test or a Mann-Whitney U-test with Bonferroni adjustment was used for paired or between group comparisons, respectively. IBM SPSS Statistics 23 (SPSS, Chicago, IL) and R software were used for statistical analysis.

## Results

### Baseline measures and a-tDCS sensations visual analog scale

There was no significant effect of condition for any of the variables measured by the POMS-short form except from vigour, although post-hoc did not show any significant differences between conditions. There were no significant differences between sessions for any of the variables measured by the wellbeing questionnaire. There were significant differences in itching, discomfort, and burning between conditions. Itching was greater after M1 (29.4, *p* < 0.001) and DLPF (19.4, *p* = 0.007) than Sham (9.2). Discomfort and burning sensation were greater only after M1 (14.7 and 21, both *p* < 0.001) compared to Sham (5.1 and 8.6) (see online resource for statistical results).

### Main analyses

**Fig. 2:**
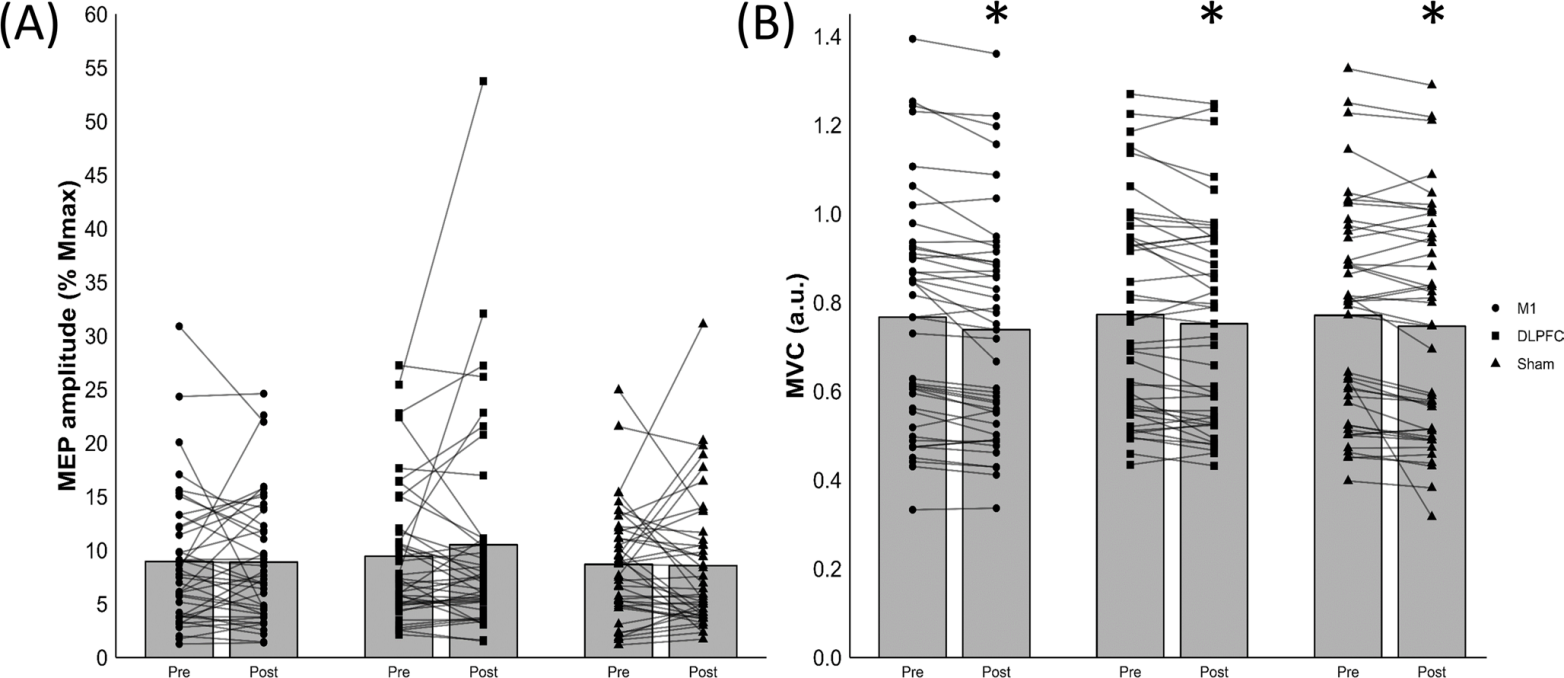
Individual (dots for M1, squares for DLPFC, and triangles for Sham) and mean values (grey bars) of (**A**) motor evoked potential amplitude (expressed as a percentage of Mmax) and (**B**) maximal voluntary isometric force, measured before (Pre) and after (Post) a-tDCS. * Statistically significant pre–post difference across all conditions (*p* < 0.05)

**Fig. 3:**
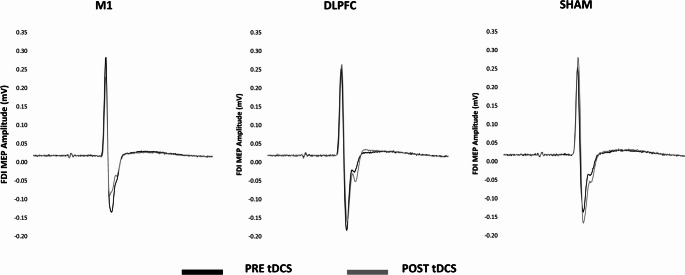
Representative single-pulse motor evoked potentials (MEPs) recorded from the first dorsal interosseous (FDI) muscle before (Pre) and after (Post) a-tDCS over M1, DLPFC and Sham. Traces correspond to the average of 20 stimuli at 120% of active motor threshold in a single participant for each condition and time point

No significant main effects or interactions were found for M_Max_, MEP/M_Max_, SICI or ICF. There was a significant main effect of time, indicating a reduction in MVC (3.2%) from pre to post, but no differences between conditions or any time × condition interaction (see online resource for statistical results) (Figs. 2 and 3).

**Fig. 4:**
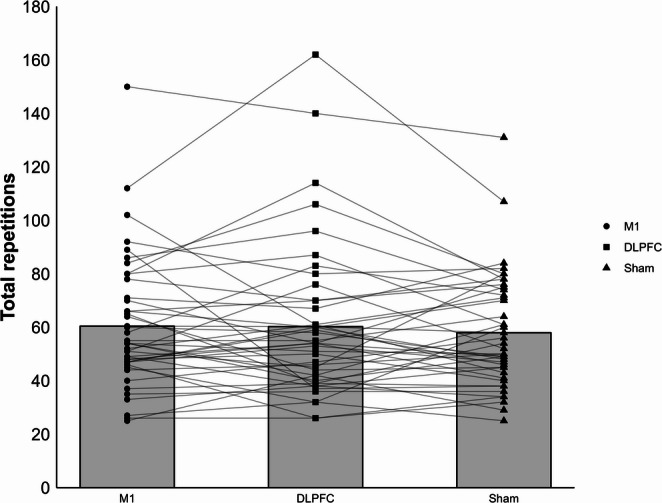
Individual (dots for M1, squares for DLPFC, and triangles for Sham) and mean values (grey bars) of the total number of repetitions completed during the repeated maximal efforts task under each stimulation condition

Regarding the RPE and the number of repetitions performed during the task (Total-Reps, or Reps-Set1, Reps-Set2 and Reps-Set3) no significant differences were observed between conditions (See Table 1 for statistical results) (Fig. 4).

**Table 1:** Mean values (± SD) and statistics results for two-way repeated measures parametric analysis of variance (ANOVA), non-parametric ANOVA-type analysis, and Friedman tests (for non-parametric variables that were only measured post stimulation)

Variable	Time	M1	DLPFC	Sham	Time	Condition	Interaction	Friedman test
M_**max**_ (mV)	Pre	5.06 ± 1.14	5.16 ± 1.32	5.02 ± 1.21	F_(1, 41)_ = 0.09, *p* = 0.76, η_p_^2^ = 0.01 [0.00, 0.13]	F_(2, 82)_ = 0.29, *p* = 0.74, η_p_^2^ = 0.01 [0.00, 0.16]	F_(1.64, 67.15)_ = 0.58, *p* = 0.53, η_p_^2^ = 0.01 [0.00, 0.18]	–
	Post	5.14 ± 1.26	5.13 ± 1.27	5.11 ± 1.17				
MEP/M_max_	Pre	0.09 ± 0.06	0.09 ± 0.06	0.09 ± 0.05	F(_1, ∞_) = 1.12, *p* = 0.29	F(_1.97, ∞_) = 0.41, *p* = 0.66	F(_1.96, ∞_) = 0.45, *p* = 0.63	–
	Post	0.09 ± 0.06	0.10 ± 0.10	0.09 ± 0.06				
SICI(% test MEP)	Pre	0.47 ± 0.39	0.41 ± 0.29	0.43 ± 0.39	F(_1, ∞_) = 0.22, *p* = 0.63	F(_1.99, ∞_) = 0.13, *p* = 0.88	F(_1.90, ∞_) = 1.00, *p* = 0.36	–
	Post	0.44 ± 0.36	0.41 ± 0.28	0.40 ± 0.27				
ICF (% test MEP)	Pre	1.43 ± 0.59	1.39 ± 0.45	1.46 ± 0.60	F(_1, ∞_) = 1.32, *p* = 0.25	F(_1.94, ∞_) = 0.87, *p* = 0.42	F(_1.96, ∞_) = 1.16, *p* = 0.31	–
	Post	1.42 ± 0.52	1.35 ± 0.78	1.44 ± 0.58				
MVC (a.u)	Pre	0.77 ± 0.26	0.77 ± 0.24	0.77 ± 0.25	F(_1, ∞_) = 30.78, *p* < 0.001	F(_1.95, ∞_) = 1.47, *p* = 0.23	F(_1.94, ∞_) = 0.11, *p* = 0.89	–
	Post	0.74 ± 0.25	0.75 ± 0.24	0.75 ± 0.26				
Total-Reps	Post	60.40 ± 24.68	60.24 ± 28.96	57.95 ± 21.64	–	–	–	χ^2^ (2) = 0.38, *P* = 0.83, W = 0.01 [0.00, 0.13]
Reps-Set 1	Post	34.74 ± 17.99	34.93 ± 18.72	32.74 ± 16.26	–	–	–	χ^2^ (2) = 0.48, *P* = 0.79, W = 0.01 [0.00, 0.14]
Reps-Set 2	Post	14.23 ± 5.37	13.02 ± 6.60	13.04 ± 5.07	–	–	–	χ^2^ (2) = 0.64, *P* = 0.73, W = 0.01 [0.00, 0.14]
Reps-Set 3	Post	11.43 ± 5.03	12.29 ± 6.31	12.17 ± 4.88	–	–	–	χ^2^ (2) = 1.18, *P* = 0.55, W = 0.01 [0.00, 0.17]
RPE(*n* = 37)	Post	14.07 ± 2.11	13.96 ± 2.24	14.12 ± 2.27	–	–	–	χ^2^ (2) = 0.15, *P* = 0.93, W = 0.01 [0.00, 0.13]

*M1* Primary motor cortex; *DLPFC* Dorsolateral prefrontal cortex; *Mmax* Maximal compound muscle action potential; *MEP* Motor-evoked potential; *SICI* short intracortical inhibition, *ICF* Intracortical facilitation; *MVC* maximum voluntary contraction; *(a.u.)* arbitrary units, *RPE*,Rating of perceived exertion

### Cluster analysis

**Fig. 5:**
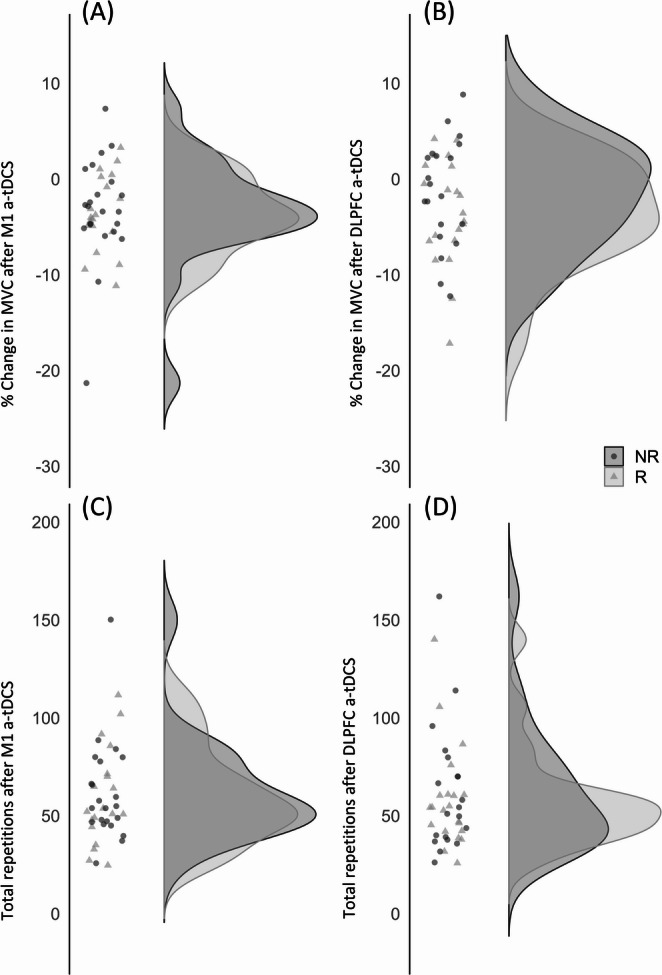
Individual and mean values (violins) of MVC (% change to pre stimulation values) and total repetitions completed by responders (R, triangles) and non-responders (NR, dots) after M1 and DLPFC a-tDCS sessions

Based on CSE response, participants were classified as 19 R and 22 NR following M1 stimulation and 21 R and 20 NR following DLPFC stimulation. The non-parametric ANOVA-type analyses did not reveal any significant main effects of time or significant time × group interactions for MVC or the number of repetitions performed during the task in either the M1 or DLPFC clusters (see online resource for statistical results and Fig. 5).

### Sex analysis

SICI was significantly reduced in males (52% of test MEP) compared to females (32% of test MEP) but no other main effects or interactions were found for MMax, MEP/MMax, SICI or ICF. MVC was significantly higher in males than in females (+ 76% on average across time and conditions *p* < 0.001). Regarding the repetitions performed during the task, females performed significantly greater number of repetitions during the second (+ 4.2 repetitions, *p* = 0.02) and third (+ 2.1 repetitions, *p* = 0.03) sets, but there was no condition main effect or interaction. For the RPE there was a sex and condition interaction but post-hoc tests did not show significant differences between conditions for any sex or between sexes differences for any condition (see online resource for statistical results).

## Discussion

In this crossover study, we investigated whether a-tDCS stimulation applied over M1 or DLPFC influences maximal handgrip force production and the ability to repeat maximal handgrip isometric contractions until a predetermined force loss threshold (−30%) in a large sample (*n* = 42). We also examined whether these potential behavioural effects were accompanied by changes in CSE, intracortical inhibition or facilitation, and perceived effort. Contrary to our hypothesis, the data show that 20 min of 2 mA a-tDCS, regardless of the stimulation area, did not influence isolated or repeated maximal force production, corticospinal or intracortical circuitry efficacy or perceived effort. The lack of effect cannot be attributed to baseline differences in mood or wellbeing, as there were no differences between conditions. Therefore, the present results suggest that M1 or DLPFC a-tDCS is not an effective technique for optimizing neuromuscular performance during isolated or repeated maximal handgrip isometric efforts.

Aside from structural factors like cross-sectional area, maximal muscle force production is determined by motor unit recruitment and discharge rate (Aagaard et al. 2020; Hortobágyi et al. 2021). Motor unit behaviour depends on synaptic inputs to the motoneuron pool from spinal afferents and supraspinal descending neurons, mainly originated in the corticospinal and reticulospinal tracts (Petersen et al. 2010; Škarabot et al. 2022). Therefore, acutely modulating the excitability of cortical neurons and interneurons projecting to the corticospinal tract could potentially modify motor unit behaviour by increasing supraspinal synaptic input. Since seminal studies demonstrated that short bouts of a-tDCS can increase CSE (Nitsche and Paulus 2000), a-tDCS has been considered a potential tool to enhance maximal force production. However, empirical evidence supporting the efficacy of a-tDCS to increase neuromuscular performance during maximal muscle contractions remains contradictory, with studies reporting increases (Hazime et al. 2017; Hummel et al. 2006; Krishnan et al. 2014; Tanaka et al. 2009, 2011; Vargas et al. 2018), decreases (Giboin and Gruber 2018; Kristiansen et al. 2022), and no effects (Grosprêtre et al. 2021; Savoury et al. 2023; Wrightson et al. 2020), as observed in the present study.

Several factors could have contributed to this controversy. For example, some studies concluding that a-tDCS increases maximal force production included clinical populations such as stroke patients (Hummel et al. 2006; Tanaka et al. 2011). It is possible that stroke patients, due to their decreased CSE in the affected hemisphere, which often correlates with poor motor function (Buetefisch et al. 2018), can acutely benefit from a-tDCS-induced increase in cortical excitability when producing force with the affected limbs. Other studies showing increased maximal force production have employed less common tests, such as toe pinch grip strength (Tanaka et al. 2009) or shoulder rotator muscle contractions (Hazime et al. 2017). Although additional studies reporting benefits have used more conventional strength assessments (e.g. elbow flexion (Krishnan et al. 2014), knee extension (Vargas et al. 2018) or even handgrip strength (Hikosaka and Aramaki 2021), their small sample sizes (*n* = 8–20), combined with the well-documented high inter-individual variability in the response to a-tDCS (López-Alonso et al. 2014) limit the robustness of these findings. In contrast, our results, obtained in a large sample of healthy young recreationally active participants (*n* = 42) show no effect of a-tDCS on maximal force production. Notably, a-tDCS was not even effective in counteracting the small but consistent reduction in MVC from pre- to post-measurements (−3%) observed across all sessions, likely resulting from repeated testing and prolonged inactivity, despite controlling for warm-up (i.e. repeating the warm-up before the post a-tDCS measurement) and for the intervals between maximal contractions (Giboin and Gruber 2018). These findings are consistent with recent crossover (Giboin and Gruber 2018; Grosprêtre et al. 2021; Kan et al. 2013; Kristiansen et al. 2022; Lampropoulou and Nowicky 2013; Savoury et al. 2023; Wrightson et al. 2020) and randomized controlled studies (Roshanzamir et al. 2024) with smaller samples (*n* = 13–27) reporting that M1 a-tDCS (with 1, 1.5 or 2 mA) does not influence maximal isometric force production or the estimated maximal dynamic force capacity (i.e. one-repetition maximum) (Alix-Fages et al. 2020).

The lack of effect of a-tDCS on maximal force production is not surprising, given the absence of changes in CSE and intracortical inhibition and facilitation, the proposed mechanisms through which a-tDCS has been proposed to improve maximal force output. Despite seminal papers showed that a-tDCS can increase CSE (Nitsche and Paulus 2000) or decrease intracortical inhibition (Stagg and Nitsche 2011), few studies have concurrently measured the effects of a-tDCS on maximal force production and CSE (Cogiamanian et al. 2007; Kristiansen et al. 2022; Lampropoulou and Nowicky 2013). In a small sample size (*n* = 6) Cogiamanian et al. (2007) found that 10 min of 1.5 mA a-tDCS increased CSE without affecting the MVC of the elbow flexors performed 60 min after a submaximal sustained contraction, which casts doubts on changes in CSE being a driving mechanism for increased neuromuscular performance during maximal contractions. However, other studies (Kristiansen et al. 2022; Lampropoulou and Nowicky 2013) have reported no changes in CSE after a-tDCS, such as Kristiansen et al. (2022) who found that 30 min of 2 mA a-tDCS did not increase neither MVC of the knee extensors or the CSE of the rectus femoris. These previous findings are consistent with our results, showing that 20 min of 2 mA a-tDCS applied over M1 or DLPFC does not influence corticospinal or intracortical excitability.

Lack of significant changes in single pulse MEP amplitude, SICI and ICF, suggest that a-tDCS did not consistently modulated the voltage-dependent ion channels (i.e. did not induce a measurable depolarisation of cortical neurons), did not reduced the concentration of gamma aminobutyric acid, and did not substantially altered the intracortical glutamatergic circuits projecting to the cortical neurons contributing to the motor evoked response. This lack of neurophysiological effects in the present study could be related to the substantial inter-individual variability in the effects of a-tDCS reported in the literature (López-Alonso et al. 2014). Therefore, effects on maximal force production could also be determined by inter-individual variability in response to a-tDCS. To test this possibility, we attempted to account for the inter-individual variability in our results by classifying participants as R or NR based on their CSE response after M1 or DLPFC a-tDCS. Even after this cluster analysis approach, our results clearly show that those participants who supposedly benefited from a-tDCS by increasing their CSE (*n* = 19 after M1 stimulation) did not experienced any effect on maximal force production. These findings coincides with a large sample-size study which did not find any changes in CSE after M1 stimulation with the same parameters used in the present study (Jonker et al. 2021). The present results not only cast doubt on the effect of a-tDCS on CSE measured at rest, but also show that even in cases where CSE increased as a consequence of a-tDCS (i.e. “responders”), maximal force production capacity did not improve. These findings reinforce the idea that slight acute variations in CSE in healthy subjects do not influence force production, which is also determined by the input from other supraspinal sources, like the reticulospinal tract (Colomer-Poveda et al. 2023; Danielson et al. 2024), or afferent input, areas that are not affected by a-tDCS.

Regarding the ability to repeat maximal isometric contractions until a predetermined force loss threshold (−30%), we hypothesized that M1 and DLPFC a-tDCS, would increase task performance through different mechanisms. An increase in CSE could lower the input required to activate the cortical neurons driving the corticospinal output to motor units (i.e. a more efficient motor command), decreasing supraspinal fatigue and allowing participants to sustain force above the required level for more repetitions (Angius et al. 2018). However, contrary to our hypothesis, our results show that 20 min of 2 mA a-tDCS applied over M1 did not increase the ability to repeat maximal contractions until a 30% loss of MVC force. The results show that no effect was present when analysing each set separately or the total number of repetitions performed across the three sets. These findings are consistent with several previous studies in which M1 stimulation failed to improve force-generating capacity during repeated muscle contractions against high-intensity dynamic or isometric loads (Giboin and Gruber 2018; Montenegro et al. 2015; Savoury et al. 2023; Workman et al. 2019, 2020). As observed for maximal force production, the results of the cluster analysis show no differences in the repetitions performed between R and NR, suggesting that acute increases in CSE of the FDI do not influence the ability to perform repeated maximal handgrip contractions.

When a-tDCS is directed to the DLPFC several studies have reported an increase in force-generating capacity during repeated high-intensity muscle contractions (Alix-Fages et al. 2019, 2020; Lattari et al. 2016, 2019, 2020; Rodrigues et al. 2022; Vieira et al. 2022). We hypothesized that a-tDCS over DLFPC would reduce RPE during the task, and allow participants to perform more maximal repetitions before reaching the predetermined force loss threshold. However, we found no effect of a-tDCS on either the total number of repetitions performed across the three sets or the number completed in each set separately. Additionally, RPE measured after finishing the task was not affected by DLPFC stimulation. Because DLPFC stimulation could interact with other remote cortical areas such as M1 (Cao et al. 2018), we applied the same classification approach (responders vs. non-responders) and tested whether there were performance differences between participants with increased and decreased CSE after DLPFC a-tDCS. However, we found no differences in any performance outcome between R and NR.

Stimulation parameters are not likely the reason for discrepancies with previous studies, since most of them used a similar stimulation configuration (15–20 min of 2 mA a-tDCS applied over the left DLPFC) (Alix-Fages et al. 2020; Lattari et al. 2019, 2020; Rodrigues et al. 2022) and these parameters have been shown to effectively modulate the DLPFC activity (Keeser et al. 2011). However, other factors related to the task could have influenced the results. For example, while most previous studies used high but submaximal muscle contraction intensities (75–80% of 1RM or MVC) (Alix-Fages et al. 2019, 2020; Lattari et al. 2016, 2019, 2020; Rodrigues et al. 2022; Vieira et al. 2022), the task in the present study required participants to perform maximal voluntary contractions from the start. This type of task could have led to a scenario where the 30% arbitrary force loss threshold was largely consequence of peripheral rather than central fatigue, as suggested by the reduction in electrically evoked twitches observed after similar protocols in the knee extensors (Giboin and Gruber 2018). This factor could have limited the capacity of a-tDCS over DLPFC to improve task performance.

The amount of muscle mass involved in the exercise could also have influenced the results. Previous studies suggest that exercises involving large muscle groups (such as squats) generate higher levels of afferent feedback (Alix-Fages et al. 2019; Rossman et al. 2014). The exercise selected in the present study (isometric handgrip contractions), which involves limited muscle mass, could have reduced the influence of afferent feedback on force production before reaching the 30% force loss threshold. Because the DLPFC integrates peripheral afferent input, this exercise selection could have limited the potential of DLPFC a-tDCS to modulate performance. Notwithstanding, despite previously mentioned factors may have contributed to the lack of effect of a-tDCS over DLPFC, it is worth noting that previous studies involved considerably smaller sample sizes than the present investigation (*n* = 10–15 per study). Therefore, replication studies using similar and different tasks are needed to clarify the effects of DLPFC a-tDCS on the ability to repeat high-intensity muscle contractions.

In exploratory analyses including sex as a between-subject factor, males showed higher MVC values whereas females completed more repetitions in the second and third sets, a pattern that is consistent with well-known sex differences in strength and fatigue resistance (Nuzzo 2023). However, no interaction between sex and a-tDCS was observed for any neuromuscular or performance variable, suggesting that the lack of a-tDCS effects on maximal and repeated handgrip performance was similar in males and females.

The present study has some limitations that could have influenced the results or restricted the generalizability of the conclusions. Although stimulation conditions were randomized and participants were blinded (including a 30-second real stimulation at 2 mA during the Sham condition) the visual analogue-scale revealed significant differences in itching, discomfort and burning sensations between the real and sham sessions. While this may have increased the likelihood that participants were able to identify the active stimulation sessions, potentially introducing expectancy-related placebo effects, the results showed no improvements in any dependent variable attributable to either a-tDCS or placebo effect. Future studies should consider the possibility that stimulation at 2 mA can be identified by participants, potentially compromising blinding procedures and inducing a placebo effect. Although standard electrode sizes were used in the present study (35cm^2^), smaller electrodes may increase the stimulation focality and require less intensity for the same stimulation density, improving the blinding process. Future studies should consider these alternative montages when the aim is to optimise focality rather than to reproduce conventional tDCS protocols. Regarding the task, as discussed, the use of maximal isometric handgrip contractions in the present study could have limited generalizability to other dynamic submaximal exercises involving larger muscle groups, such as those commonly prescribed during resistance training (e.g. squats, bench press). Additionally, in the present study we chose the FDI muscle to determine the M1 anodal electrode position. The FDI contributes synergistically to force production during a power grip by acting in opposition to the thumb, leading to corticospinal excitability increases during power grip contractions (Datta et al. 1989; Flament et al. 1993; Reilly and Mercier 2008). However, the FDI is not the main muscle generating force during a power grip, which may have limited the effect of a-tDCS in the tasks tested in this study. Nevertheless, there is a substantial overlap between the cortical representations of the FDI and the flexor digitorum superficialis (70–85%) (Jin et al. 2023), one on the main contributors to power grip force. Therefore, given this overlap and the size of the electrodes used in the present study (35cm2), it is unlikely that cortical neurons projecting to the finger flexors did not receive stimulation by our M1 montage. Finally, the present results should be interpreted within the context of the study population (healthy, recreationally active young adults), taking in to account that the effects of a-tDCS may differ in clinical populations (e.g. stroke) (Hummel et al. 2006; Tanaka et al. 2011) or in highly trained adults (Maudrich et al. 2022).

Collectively, the data show that a-tDCS over M1 or DLPFC does not improve maximal handgrip force production or the ability to repeat maximal handgrip isometric contractions until a predetermined force loss threshold (−30%) in a large sample size (*n* = 42). This lack of performance effects was not accompanied by cortical or corticospinal modulation, nor by changes in perceived effort. These results suggest that M1 or DLPFC a-tDCS may not be an effective performance-enhancing tool to improve maximal force or neuromuscular endurance during high-intensity repeated muscle contractions.

## Supplementary Information

Below is the link to the electronic supplementary material.

Supplementary Material 1

## Data Availability

The datasets generated during and/or analysed during the current study are available from the corresponding author on reasonable request.

## Abbreviations

a-tDCS: Anodal transcranial direct current stimulation
CSE: Corticospinal excitability
DLPFC: Dorsolateral prefrontal cortex
EMG: Electromyography
FDI: First dorsal interosseous
ICF: Intracortical facilitation
M1: Primary motor cortex
MEP: Motor evoked potential
M_Max_: Maximal compound muscle action potential
MVC: Maximal voluntary contraction
NR: No responders
POMS: Profile of Mood States
R: Responders
RM-ANOVA: Repeated measures analysis of variance
RMS: Root mean square
rMT: Resting motor threshold
RPE: Rate of perceived exertion
SICI: Short interval intracortical inhibition
tDCS: Transcranial direct current stimulation
TMS: Transcranial magnetic stimulation
