# Supplementary material for: Acute effects of anodal transcranial direct current stimulation on maximal voluntary force, repeated maximal isometric contractions and corticospinal excitability in healthy young adults
Source: Eur J Appl Physiol. 2026 Mar 2;126(6):3519–32. doi: 10.1007/s00421-026-06186-w (PMC13287258; doi:10.1007/s00421-026-06186-w)
Supplement: Supplementary file 1 — Supplementary Material 1 [file 421_2026_6186_MOESM1_ESM.docx]

**Table 1.** Baseline POMS and readiness and visual analog scale mean values (±SD) and statistics results for the one-way repeated measures analysis of variance (RM-ANOVA) or Friedman test depending on data distribution.

| **Variable** | **M1** | **DLPFC** | **Sham** | **Friedman or ANOVA test** |
| --- | --- | --- | --- | --- |
| **Itching** (n=36) | 29.44±24.72 | 19.44±20.17 | 9.17±11.98 | χ^2^ (2) = 18.74, P < 0.001 |
| **Discomfort**  (n=36) | 14.71±17.36 | 10.57±15.84 | 5.14±7.02 | χ^2^ (2) = 11.27, P = 0.004 |
| **Burning**  (n=36) | 20.97±25.91 | 11.39±16.41 | 8.61±15.01 | χ^2^ (2) = 7.59, P = 0.023 |
| **POMS** | | | | |
| **Tension** | 3.07±2.99 | 3.02±3.50 | 2.54±2.85 | χ^2^ (2) = 0.59, P = 0.75 |
| **Anger** | 1.29±2.10 | 1.78±3.61 | 1.85±2.93 | χ^2^ (2) = 1.50, P = 0.47 |
| **Depression** | 1.46±2.29 | 1.56±2.26 | 1.85±2.26 | χ^2^ (2) = 1.88, P = 0.38 |
| **Fatigue** | 4.88±2.85 | 4.80±3.57 | 6.10±4.15 | χ^2^ (2) = 0.51, P = 0.77 |
| **Vigor** | 10.88±2.90 | 11.24±3.35 | 9.76±3.69 | F_(2, 80)_ = 3.30, p = 0.04, η_p_^2^ = 0.08 |
| **Confusion** | 14.83±2.61 | 15.21±3.11 | 14.90±2.83 | F_(2, 80)_ = 0.41, p = 0.66, η_p_^2^ = 0.01 |
| **Wellbeing questionnaire** | | | | |
| **Sleep hours** | 6.85±1.06 | 7.10±1.02 | 6.79±1.12 | χ^2^ (2) = 5.31, P = 0.07 |
| **Sleep quality** | 6.27±1.25 | 6.61±1.14 | 6.10±1.39 | χ^2^ (2) = 4.71, P = 0.10 |
| **Muscular pain** | 2.49±1.26 | 2.95±1.90 | 3.05±1.87 | χ^2^ (2) = 1.67, P = 0.43 |
| **Fatigue** | 3.02±1.23 | 3.17±1.50 | 3.71±2.12 | χ^2^ (2) = 1.31, P = 0.52 |
| **Stress** | 2.90±1.73 | 3.12±2.28 | 2.88±1.81 | χ^2^ (2) = 0.05, P = 0.97 |

Abbreviations: M1, Primary motor cortex; DLPFC, Dorsolateral prefrontal cortex.

**Table 2.** Cluster analysis. Mean values (±SD) and statistics results for non-parametric ANOVA-type analysis (f1.ld.f1) with time and group (Responder, No Responder) as factors for M1, DLPFC and Sham conditions. Responder and no responder were specifically assessed for each condition based on positive (R, responder) or negative (NR, no responder) changes in CSE after real or sham a-tDCS stimulation period.

| **Condition** | **Outcome** | **Cluster (n)** | **Pre** | **Post** | **Set1** | **Set2** | **Set3** | **np-anova: Time** | **np-anova: Group** | **np-anova: Interaction** |
| --- | --- | --- | --- | --- | --- | --- | --- | --- | --- | --- |
| M1 | MVC | R (19) | 0.75±0.28 | 0.72±0.28 | ---- | ---- | ---- | F(_1, ∞_) = 20.04, p < 0.001 | F(_1, ∞_) = 0.14, p = 0.69 | F(_1, ∞_) = 0.01, p = 0.94 |
|  |  | NR (22) | 0.77±0.25 | 0.75±0.23 | ---- | ---- | ---- |  |  |  |
|  | Repetitions | R (19) | ---- | ---- | 33.26±16.01 | 14.53±6.29 | 11.74±4.91 | F(_1.98, ∞_) = 179.70, p < 0.001 | F(_1, ∞_) = 0.01, p = 0.93 | F(_1.98, ∞_) = 0.14, p = 0.87 |
|  |  | NR (22) | ---- | ---- | 36.36±20.11 | 14.09±4.67 | 11.31±5.30 |  |  |  |
| DLPFC | MVC | R (21) | 0.78±0.25 | 0.75±0.24 | ---- | ---- | ---- | F(_1, ∞_) = 5.67, p = 0.02 | F(_1, ∞_) = 0.02, p = 0.89 | F(_1, ∞_) = 0.96, p = 0.33 |
|  |  | NR (20) | 0.75±0.23 | 0.75±0.24 | ---- | ---- | ---- |  |  |  |
|  | Repetitions | R (21) | ---- | ---- | 33.01±16.61 | 12.62±6.15 | 12.57±4.91 | F(_1.96, ∞_) = 132.31, p < 0.001 | F(_1, ∞_) = 3.12, p = 0.95 | F(_1.98, ∞_) = 1.28, p = 0.28 |
|  |  | NR (20) | ---- | ---- | 36.85±21.39 | 13.55±7.32 | 11.95±7.25 |  |  |  |

Abbreviations: np, non-parametric; M1, Primary motor cortex; DLPFC, Dorsolateral prefrontal cortex; MVC, maximum voluntary contraction.

| **Variable** | **Sex** | **Time** | **M1** | **DLPFC** | **Sham** | **Sex** | **Time** | **Sex*Time** | **Condition** | **Sex*Condition** | **Time*Condition** | **Sex*Time*Condition** |
| --- | --- | --- | --- | --- | --- | --- | --- | --- | --- | --- | --- | --- |
| **M_max_ (mV)** | Female | Pre | 5.13±1.29 | 5.25±1.50 | 5.06±1.30 | F(_1, 40_) = 0.34,  p = 0.56,  η_p_^2^ = 0.01 | F(_1, 40_) = 0.87, p = 0.77,  η_p_^2^ = 0.01 | F(_1, 40_) = 0.01, p = 0.96,  η_p_^2^ = 0.01 | F(_1, 80_) = 0.29, p = 0.75,  η_p_^2^ = 0.01 | F(_1, 80_) = 0.11, p = 0.90,  η_p_^2^ = 0.01 | F(_2, 64.05_) = 0.72, p = 0.46,  η_p_^2^ = 0.02 | F(_2, 64.05_) = 0.91, p = 0.39,  η_p_^2^ = 0.02 |
|  |  | Post | 5.30±1.41 | 5.33±1.28 | 5.23±1.29 |  |  |  |  |  |  |  |
|  | Male | Pre | 5.01±1.03 | 5.08±1.19 | 5.00±1.17 |  |  |  |  |  |  |  |
|  |  | Post | 5.01±1.03 | 4.97±1.27 | 5.02±1.08 |  |  |  |  |  |  |  |
| **MEP/M_max_** | Female | Pre | 0.10±0.06 | 0.11±0.07 | 0.10±0.06 | F(_1, ∞_) = 2.98,  p = 0.08 | F(1_, ∞_) = 0.97,  p = 0.32 | F(1_, ∞_) = 0.06,  p = 0.80 | F(_2, ∞_) = 1.10,  p = 0.57 | F(_2, ∞_) = 2.10,  p = 0.35 | F(_2, ∞_) = 0.95,  p = 0.62 | F(_2, ∞_) = 1.11,  p = 0.57 |
|  |  | Post | 0.10±0.06 | 0.14±0.13 | 0.10±0.07 |  |  |  |  |  |  |  |
|  | Male | Pre | 0.08±0.06 | 0.08±0.05 | 0.07±0.04 |  |  |  |  |  |  |  |
|  |  | Post | 0.08±0.05 | 0.07±0.05 | 0.08±0.05 |  |  |  |  |  |  |  |
| **SICI (% test MEP)** | Female | Pre | 0.34±0.36 | 0.35±0.33 | 0.29±0.22 | F(_1, ∞_) = 10.35,  p < 0.01 | F(1_, ∞_) = 0.24,  p = 0.62 | F(1_, ∞_) = 0.32,  p = 0.57 | F(_1.99, ∞_) = 0.08,  p = 0.92 | F(_1.99, ∞_) = 1.99,  p = 0.11 | F(_1.95, ∞_) = 1.03,  p = 0.36 | F(_1.95, ∞_) = 0.62,  p = 0.53 |
|  |  | Post | 0.28±0.26 | 0.32±0.25 | 0.33±0.21 |  |  |  |  |  |  |  |
|  | Male | Pre | 0.59±0.37 | 0.46±0.26 | 0.54±0.47 |  |  |  |  |  |  |  |
|  |  | Post | 0.57±0.38 | 0.50±0.28 | 0.47±0.30 |  |  |  |  |  |  |  |
| **ICF**  **(% test MEP)** | Female | Pre | 1.30±0.53 | 1.39±0.45 | 1.36±0.56 | F(_1, ∞_) = 1.66,  p = 0.20 | F(1_, ∞_) = 1.25,  p = 0.26 | F(1_, ∞_) = 0.02,  p = 0.88 | F(_1.98, ∞_) = 0.67,  p = 0.51 | F(_1.98, ∞_) = 1.42,  p = 0.24 | F(_1.95, ∞_) = 1.12,  p = 0.32 | F(_1.95, ∞_) = 0.08,  p = 0.92 |
|  |  | Post | 1.29±0.55 | 1.27±0.41 | 1.31±0.55 |  |  |  |  |  |  |  |
|  | Male | Pre | 1.54±0.62 | 1.39±0.47 | 1.56±0.64 |  |  |  |  |  |  |  |
|  |  | Post | 1.54±0.48 | 1.44±1.01 | 1.56±0.59 |  |  |  |  |  |  |  |
| **MVC (a.u)** | Female | Pre | 0.53±0.09 | 0.55±0.07 | 0.55±0.09 | F(_1, ∞_) = 115.56, p < 0.001 | F(_1, ∞_) = 32.08,  p < 0.001 | F(_1.93, ∞_) = 0.12,  p = 0.73 | F(_1.93, ∞_) = 1.66, p = 0.19 | F(_1.93, ∞_) = 0.53,  p = 0.58 | F(_1.93, ∞_) = 0.09,  p = 0.90 | F(_1.93, ∞_) = 0.40,  p = 0.66 |
|  |  | Post | 0.51±0.08 | 0.54±0.07 | 0.52±0.09 |  |  |  |  |  |  |  |
|  | Male | Pre | 0.96±0.18 | 0.95±0.16 | 0.96±0.17 |  |  |  |  |  |  |  |
|  |  | Post | 0.92±0.18 | 0.93±0.16 | 0.93±0.20 |  |  |  |  |  |  |  |
| **Total-Reps** | Female | ---- | 68.9±28.7 | 66.4±34.6 | 67.3±24.9 | F(_1, ∞_) = 3.79, p = 0.05 | ---- | ---- | F(_1.99, ∞_) = 0.57, p = 0.56 | F(_1.99, ∞_) = 1.20, p = 0.30 | ---- | ---- |
|  | Male |  | 53.4±18.6 | 55.1±22.8 | 50.2±15.1 |  |  |  |  |  |  |  |
| **Reps-Set 1** | Female | ---- | 30.6±12.9 | 32.7±16.7 | 27.3±12.8 | F(_1, ∞_) = 3.25, p = 0.07 | ---- | ---- | F(_1.98, ∞_) = 0.40, p = 0.67 | F(_1.98, ∞_) = 1.94, p = 0.14 | ---- | ---- |
|  | Male |  | 39.8±22.0 | 37.6±21.1 | 39.3±17.9 |  |  |  |  |  |  |  |
| **Reps-Set 2** | Female | ---- | 16.5±2.9 | 15.2±8.9 | 14.1±6.1 | F(_1, ∞_) = 5.08, p = 0.02 | ---- | ---- | F(_1.98, ∞_) = 2.76, p = 0.06 | F(_1.98, ∞_) = 2.19, p = 0.11 | ---- | ---- |
|  | Male |  | 12.3±5.3 | 11.2±2.9 | 12.2±3.9 |  |  |  |  |  |  |  |
| **Reps-Set 3** | Female | ---- | 12.6±5.0 | 13.6±6.6 | 13.9±5.0 | F(_1, ∞_) = 4.47, p = 0.03 | ---- | ---- | F(_1.98, ∞_) = 0.72, p = 0.49 | F(_1.98, ∞_) = 0.47, p = 0.62 | ---- | ---- |
|  | Male |  | 10.5±4.9 | 11.2±6.0 | 10.7±4.4 |  |  |  |  |  |  |  |
| **RPE**  (n=37) | Female | ---- | 14.1±2.1 | 13.7±2.4 | 14.3±2.3 | F(_1, ∞_) = 0.21, p = 0.65 | ---- | ---- | F(_1.96, ∞_) = 0.12, p = 0.88 | F(_1.96, ∞_) = 3.03, p = 0.049 | ---- | ---- |
|  | Male |  | 14.0±2.2 | 14.1±2.1 | 14.0±2.3 |  |  |  |  |  |  |  |

**Table 3.** Sex analysis. Mean (±SD) values and statistical results from RM-ANOVA and non-parametric ANOVA-type models including sex as a between-subject factor for repeated-measures outcomes (sex × time × condition) and for single post-stimulation measures (sex × condition).

Abbreviations: M1, Primary motor cortex; DLPFC, Dorsolateral prefrontal cortex; Mmax, Maximal compound muscle action potential; MEP, Motor-evoked potential; SICI, short intracortical inhibition, ICF, Intracortical facilitation; MVC, maximum voluntary contraction; (a.u.), arbitrary units, RPE, Rating of perceived exertion.

Table 4. Relative treatment effects of the non-parametric ANOVA-type models of the main analyses (RTE) with 95% confidence intervals for each level of the main factors (Time and Condition).

| **Variable** | **Factor** | **Level** | **RTE (95%CI)** |
| --- | --- | --- | --- |
| MVC | Time | Pre | 0.52 (0.43-0.60) |
| MVC | Time | Post | 0.48 (0.40-0.57) |
| MVC | Condition | Sham | 0.50 (0.39-0.61) |
| MVC | Condition | M1 | 0.49 (0.38-0.60) |
| MVC | Condition | DLPFC | 0.51 (0.40-0.62) |
| MEP/M_Max_ | Time | Pre | 0.51 (0.42-0.60) |
| MEP/M_Max_ | Time | Post | 0.49 (0.40-0.58) |
| MEP/M_Max_ | Condition | Sham | 0.49 (0.38-0.60) |
| MEP/M_Max_ | Condition | M1 | 0.50 (0.39-0.61) |
| MEP/M_Max_ | Condition | DLPFC | 0.51 (0.41-0.62) |
| SICI | Time | Pre | 0.50 (0.41-0.58) |
| SICI | Time | Post | 0.50 (0.42-0.59) |
| SICI | Condition | Sham | 0.49 (0.38-0.60) |
| SICI | Condition | M1 | 0.50 (0.40-0.61) |
| SICI | Condition | DLPFC | 0.51 (0.40-0.61) |
| ICF | Time | Pre | 0.51 (0.42-0.60) |
| ICF | Time | Post | 0.49 (0.40-0.58) |
| ICF | Condition | Sham | 0.51 (0.41-0.62) |
| ICF | Condition | M1 | 0.51 (0.41-0.62) |
| ICF | Condition | DLPFC | 0.47 (0.36-0.58) |

Abbreviations: MVC, maximum voluntary contraction; M1, Primary motor cortex; DLPFC, Dorsolateral prefrontal cortex; MEP, Motor-evoked potential; Mmax, Maximal compound muscle action potential; SICI, short intracortical inhibition, ICF, Intracortical facilitation; RPE, Rating of perceived exertion.
